# Dormant cancer cells: programmed quiescence, senescence, or both?

**DOI:** 10.1007/s10555-022-10073-z

**Published:** 2023-01-04

**Authors:** Kevin Truskowski, Sarah R. Amend, Kenneth J. Pienta

**Affiliations:** 1 Brady Urological Institute, Johns Hopkins School of Medicine, 600 North Wolfe St, Baltimore, MD USA; 2 Cellular and Molecular Medicine Graduate Training Program, Johns Hopkins School of Medicine, 1830 E. Monument St. Suite 20103, Baltimore, MD 21205 USA; 3 Cancer Ecology Center, Johns Hopkins School of Medicine, 600 North Wolfe St, Baltimore, MD USA

**Keywords:** Cancer, Dormancy, Quiescence, Senescence, Metastasis, Cell cycle

## Abstract

Metastasis is the overwhelming driver of cancer mortality, accounting for the majority of cancer deaths. Many patients present with metastatic relapse years after eradication of the primary lesion. Disseminated cancer cells can undergo a durable proliferative arrest and lie dormant in secondary tissues before reentering the cell cycle to seed these lethal relapses. This process of cancer cell dormancy remains poorly understood, largely due to difficulties in studying these dormant cells. In the face of these challenges, the application of knowledge from the cellular senescence and quiescence fields may help to guide future thinking on the study of dormant cancer cells. Both senescence and quiescence are common programs of proliferative arrest that are integral to tissue development and homeostasis. Despite phenotypic differences, these two states also share common characteristics, and both likely play a role in cancer dormancy and delayed metastatic relapse. Understanding the cell biology behind these states, their overlaps and unique characteristics is critical to our future understanding of dormant cancer cells, as these cells likely employ some of the same molecular programs to promote survival and dissemination. In this review, we highlight the biology underlying these non-proliferative states, relate this knowledge to what we currently know about dormant cancer cells, and discuss implications for future work toward targeting these elusive metastatic seeds.

## Introduction 

Cancer is generally described as a disease of uncontrolled cellular proliferation. This growth phenotype is well characterized and driven by defined hallmarks that include the induction of sustained proliferative signaling (e.g., gain-of-function mutations in oncogenes), evasion of growth suppressive signals (e.g., loss-of-function mutations in tumor suppressors), and resistance against death signals (e.g., avoidance of apoptotic signaling and immune destruction) [1–3]. While understanding these hallmarks is critical, cancer is a complex adaptive system that is more than a simple sum of these characteristics. For example, if cancer was truly a disease simply of unattenuated proliferation, we might predict that all tumors would be eradicated by radiation and chemotherapy regimens that target such proliferative cells. In fact, however, the majority of cancers kill patients after such treatments have failed. How and why these treatments fail varies based on treatment, tumor type, genetic background, etc., but one mechanism that has been shown to play a role is strategic, regulated proliferation arrest in tumor cells. This process, which is critical in organismal development, may also be critical in tumorigenesis and cancer progression, especially with regard to therapy resistance, tumor dormancy, and delayed recurrence.

Tumor dormancy refers to how a cancer cell or small cluster of cells may remain micrometastatic for years or decades before outgrowth. It remains a topic rife with urgent unanswered questions for the field. An appreciation of the distinct mechanisms of proliferative arrest that occur in cancer may help guide our understanding of the mechanisms by which a proliferative cancer cell may halt division to resist treatment or to metastasize, remain dormant at a distant site then regain proliferative potential to seed lethal recurrence. As the field works to better define the fundamental characteristics of these dormant cancer cells, it is important to compare what we know in this field to what we know surrounding the two common forms of proliferative arrest described in cancer and normal physiology-senescence and quiescence. Are the same programs that drive arrest in these contexts involved in this process of tumor dormancy? If so, how?

## Cancer dormancy

Metastatic recurrence after eradication of the primary tumor is common and often occurs years after initial remission. Described in most common solid cancers, including breast, prostate, lung, and colon cancers, as well as in hematological cancers [4], this process of late recurrence has been observed for centuries but is only now beginning to be better characterized. The term dormancy was first applied to the field in 1934 by Rupert Willis when he described late metastases in patients who had no evidence of local recurrence, proposing that these cancer cells entered the secondary tissue and “must have lain dormant” [5]. Geoffrey Hadfield postulated in 1954 that these late recurrences are driven by cells that had undergone a “temporary mitotic arrest” [6].

Dissemination from the primary tumor, once thought to be only a late-stage event, is now understood to also occur early in tumorigenesis [4, 7]. Some of the best evidence for early dissemination and subsequent dormancy comes from studies of patients who received organ transplants from people who either had no previous cancer diagnosis or had been cured for over 10 years. Patients receiving different organs from the same donor went on to develop tumors, and some eventually succumbed to the disease (reviewed in [4]). Disseminated tumor cells (DTCs) from early primary lesions apparently undergo a period of stasis during which no clinically detectable tumors arise in secondary sites for years or decades, much longer than would be predicted in the absence of a dormant period.

These periods of dormancy have been described previously as driven by two related processes. The first is cellular or cell-intrinsic dormancy, where a solitary DTC or small cluster of DTCs are held in a state of proliferative arrest at the secondary site. These dormant DTCs may then progress to a state of “tumor mass” dormancy, an instance where a metastatic lesion is made up of proliferative cells, but the tumor is held below a clinically detectible size threshold by suppressive mechanisms such as lack of blood and nutrient supply (“angiogenic dormancy”) or by active surveillance by the immune system (“immune-mediated dormancy”) [8, 9]. Due to difficulties in preclinical models for tracking dormant cells *in vivo*, as well as our inability to study undetectable metastases in patients, rigorous studies into the identities and characteristics of these dormant tumor cells remain challenging. Despite this, many studies have begun to uncover some of these unknowns in the past two decades. These works are well reviewed elsewhere, including [8–18], and more recent studies are highlighted below.

Dormancy appears to be an adaptive strategy that cancer cells employ in response to environmental or therapeutic stressors, and these cells are a reservoir for delayed relapse. Tumor dormancy is a central process in cancer progression and a potential target for new therapies, but it remains to be fully understood. Currently, the field is clouded by the use of various terms with imprecise and overlapping definitions. “Quiescent,” “slow-cycling,” “drug-tolerant persister,” “reversibly senescent,” “cancer stem cell-like”, and other terms have all been equated to”dormant” cancer cells. As illustrated by these various designations, dormant cancer cells share characteristics with other cells undergoing proliferative arrest, namely quiescent and senescent cells. Exploring the roles of quiescence and senescence in normal physiology and in cancer will advance our understanding of dormancy and delayed relapse. Increasing our understanding of this phase of the disease may elucidate new paradigms for the treatment of patients, with the potential to prevent metastatic outgrowth, the overwhelming driver of cancer mortality.

## Cellular senescence in cancer

The process of cellular senescence originally described the limited replicative lifespan of human cells grown *in vitro*, now more accurately referred to as ‘replicative senescence’ and known to occur *in vivo*. Hayflick demonstrated in the 1960s that human fibroblasts grown *in vitro* undergo proliferative arrest after a set number of divisions (now termed the ‘Hayflick limit’) [19, 20]. He considered this a terminal fate, an assertion that has been challenged by more recent data. It is now known that this process of replicative senescence is driven by loss of telomeres and persistent DNA damage signaling [21–25]. Although these studies were performed in normal cells, it is now clear that senescence can be induced in cancer cells in response to various stressors. These other processes that converge on a similar senescent phenotype are of interest in cancer and are now described as stress-induced premature senescence (SIPS); premature in the sense that the phenotype is not driven by the cells nearing the end of their replicative lifespan but by aberrant oncogene expression (oncogene-induced senescence, OIS) [26–30] or cytotoxic stresses from chemotherapy (therapy-induced senescence, TIS) [31]. Telomere shortening is not implicated in all cases, although importantly, all three of these processes are largely driven by a persistent DNA damage response. DNA damage arises due to telomere shortening, replicative stress, or direct DNA damaging agents in replicative, oncogene-induced, and therapy-induced senescence, respectively (Table 1) [25, 30, 32, 33].

**Table 1:** Characteristics of quiescence and senescence

	Quiescence	Senescence
Driver	*In vitro: *serum deprivation, contact inhibition, loss of adhesion	Telomere shortening (RS), replication stress (OIS), DNA-damaging agents (TIS) resulting in persistent DNA damage response (DDR)
	*In vivo: *niche factors, lack of mitogens, loss of cell/cell or cell/ECM contact	
Key markers	**↑**p27, **p21**; S, G2/M cyclin –	Enlarged, flattened, granular morphology; B-gal+; **↑**p16, **p21**; SAHF; SASP
Cell cycle state	G0	G1, G2^?^
Molecular regulators	**CDKis, Rb/E2F switch**	DDR components, **CDKis, Rb/E2F switch**
Reversible?	Yes	Possibly
Resistance to cell death	Resistant to agents that target proliferative cells; immune evasive	Resistant to agents that target proliferative cells; upregulated anti-apoptotic factors

*shared characteristics bolded

In the 60 years since Hayflick’s initial description of cellular senescence, the definition of a senescent cell has evolved beyond describing a simple proliferative arrest to include morphological changes such as enlargement and flattening of cells; activation of tumor suppressor networks such as p16/Rb or p53/p21 that arrest cells in G1 or G2 phase [34]; an increase in senescence-associated β-galactosidase (SA-β-Gal) activity; chromatin structure changes, including the presence of senescence-associated heterochromatin foci (SAHF); and changes in the transcriptome that promote the secretion of a collection of growth factors, cytokines, chemokines, and proteases known as the senescence-associated secretory phenotype (SASP) (Table 1) [35, 36]. Evidence for the roles of cellular senescence in normal physiology, namely in organismal development, tissue patterning, and wound healing processes, has also been uncovered [37–45], along with roles in pathophysiology related to aging [46].

**Fig. 1:**
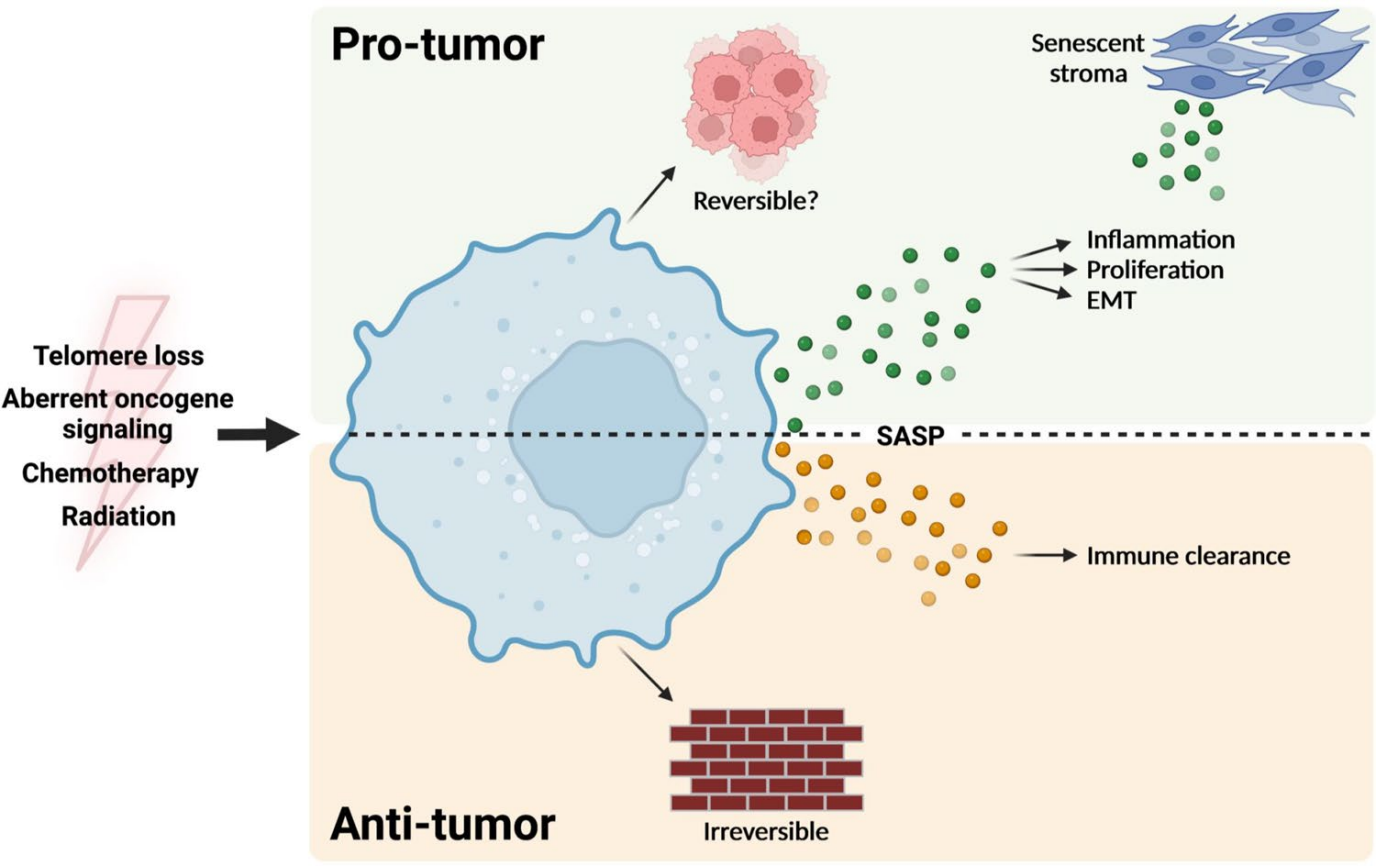
The pro-tumor and anti-tumor aspects of senescence. Telomere loss, oncogene signaling, or therapies that target proliferative cells (e.g., chemotherapy and radiation) can all drive a cancer cell to senescence. Senescence is classically described as a permanent state and would therefore impede tumor formation. Recent data suggest that senescence may be reversible, in which case, it could be an adaptive mechanism for therapy resistance. The SASP plays both pro- and anti-tumor roles. In certain contexts, it signals to the immune system to promote clearance of cancer cells by T cells and NK cells. In other contexts, factors secreted by senescent tumor or stromal cells can promote inflammation, proliferation, and EMT in a paracrine fashion. Created with BioRender.com

Importantly, evidence has also accumulated pointing to roles for senescence during tumorigenesis [3, 22, 25, 30, 47, 48]. At first glance, the induction of persistent proliferative arrest appears to be beneficial for halting tumor growth. Indeed, much has been written that supports this view, hypothesizing, for example, that treating patients with lower doses of chemotherapies may be able to induce senescence and tumor growth arrest without the toxicities associated with the higher doses of these drugs that are utilized currently [49]. As with most things in cancer, however, senescence appears to be a double-edged sword, and several groups have reported findings describing the protumor functions of senescent cells (Fig. 1) [50].

### Antitumor aspects of senescence

The most intuitive case of the involvement of senescence (a form of proliferative arrest) in cancer (a disease of hyperproliferation) is its role as an opposing force to tumor growth. Several groups have reported roles for senescence in protecting against transformation and tumor initiation. Studies in various tumor types have documented the presence of senescent cells in premalignant lesions, which are then rare once the lesions have progressed to malignancy [51–55], implying that senescent cells present a barrier for tumorigenesis. Although these studies are correlational, they are largely in agreement with the data on oncogene-induced senescence in normal or premalignant cells in which the senescence response halts the proliferation of cells at risk of transformation, preventing tumor formation. This response was first described in the context of HRAS^V12^ expression and has since been shown to occur in response to gain-of-function mutations in other oncogenes, including *BRAF*, *AKT*, and the cell cycle regulators *E2F1* and *cyclin E*. Similarly, loss-of-function mutations in the tumor suppressors *PTEN* and *NF1* can trigger the same response *in vitro* and *in vivo* [28, 51–57]. Moreover, senescent cells are characterized by the SASP, which contains a host of immune-modulating cytokines and chemokines. Similar to its roles in tissue development and patterning, the SASP may induce immune-mediated clearance of precancerous cells, which may act to further prevent tumor initiation (Fig. 1) [58–60]. Cells that avoid senescence, successfully transform, and go on to form detectable tumors will inevitably be treated with chemotherapy or radiation, both of which have been shown to drive TIS, halting tumor growth and promoting regression through immune-mediated clearance [10, 60, 61]. Although these mechanisms of senescence are intuitively tumor antagonistic, the same mechanisms may actually be tumor promoting depending on the context.

### Pro-tumor aspects of senescence

Although the role of cellular senescence in organismal aging is not entirely clear, it is now well understood that aged organisms accumulate senescent cells in a variety of tissues. As cancer is often described as a ‘disease of aging,’ with incidence rates increasing dramatically with age, it seems likely that senescence and cancer are related. In fact, Rudolf Virchow touched on this over 100 years before the discovery of cellular senescence with his ‘irritation theory’ from 1858 [62], where he proposed that chronic irritation and inflammation are drivers of tumorigenesis. Harold Dvorak subsequently characterized tumors as ‘wounds that do not heal’. Senescent cells, known to play a role in wound healing and inflammation through the action of the SASP, induce remodeling of the microenvironment that, while beneficial for wound healing, may contribute to the formation of neoplasia [63, 64]. In this way, the same process that halts proliferation of cells at risk for transformation may paradoxically drive tumor initiation. Halazonetis and colleagues proposed that oncogene-induced replication stress and subsequent DNA damage may drive not only OIS but also the genomic instability critical for the accumulation of mutations in cells that promote cancer development [47]. While it is unclear under which circumstances cells would be induced to undergo senesce versus undergoing transformation and tumor formation, it is likely that the same upstream events can promote both fates, further linking senescence to cancer development.

Much of the research on senescence in cancer has focused on elucidating the roles of aging stromal components in cancer progression. Senescent fibroblasts and other stromal components have been shown to induce proliferation, motility, and angiogenesis in cancer cells, largely through the autocrine and paracrine actions of SASP components (Fig. 1) [65–68]. In the same way, however, components of the SASP secreted from a cancer cell that has undergone OIS or TIS may also impact other cancer cells in a paracrine fashion and impact tumor progression and response to therapy. In vitro, media conditioned by cells that have undergone TIS can stimulate proliferation, induction of hallmark epithelial-mesenchymal transition (EMT) markers, and increased invasiveness [69]. Additionally, responses to radiation and chemotherapy can be affected by cancer cells undergoing TIS. Senescence in response to these therapies can inhibit apoptosis and even lead to faster relapse than in tumors unable to undergo senescence [60, 70, 71].

### Cellular senescence: links to dormancy

The identity of dormant cancer cells remains elusive in general, and therefore, studies directly identifying dormant cancer cells as senescent are scarce. The definition of a ‘dormant cancer cell’ requires that the cell be able to reawaken and proliferate to repopulate a tumor. Therefore, by the classical definition of senescence as permanent proliferative arrest, cells that have undergone senescence could not contribute to delayed relapse as a dormant seed. For a senescent cancer cell and a dormant cancer cell to be one in the same hinges on the controversial claim that senescence is reversible. Data on the reversibility of senescence do exist, although none are definitive, and all are context dependent (Fig. 1) [9, 10, 72–78].

Some of the studies linking senescence to tumor initiation could also be relevant to the reawakening and colonization of dormant cells. This may be especially true in TIS, as dormant tumor cells are relevant to disease progression during and after treatment. Senescent cells persisting after therapy would certainly be a component contributing to minimal residual disease (MRD). One study, for example, directly links a treatment-induced ‘senescence-like state’ to dormancy in the context of EGFR targeted therapy in *EGFR-*mutant non-small-cell lung cancer [79].

If senescence is truly reversible in certain contexts, then senescent cells could plausibly be related to dormant cells based on several shared characteristics. First, the duration of dormancy seen in patients and in preclinical models lends itself to the idea that these dormant cells have undergone a durable arrest, much like senescent cells. Other types of arrested cells, such as those in quiescence, could presumably resume proliferation more quickly than what is seen in dormancy models, although progression of a solitary dormant cell to so-called ‘tumor-mass dormancy’ controlled by the immune system could account for this time before detectable relapse. Senescent cells, like dormant cancer cells, are also refractory to apoptosis in response to therapies that target proliferative cells. This resistance is critical for dormant cells to survive long enough in a patient undergoing treatment to go on to seed relapse. Induction of a reversible senescence program could represent an adaptive mechanism for cells in response to chemotherapeutic stress.

In addition to the senescent cell playing the role of the dormant seed for delayed metastasis, a possibly less controversial role for senescent cells in dormancy and delayed relapse could be due to paracrine signaling from the senescent cells’ SASP. As has been proposed in the initial transformation and formation of a primary tumor, perhaps signals from a senescent tumor cell at the metastatic site remodel the microenvironment to drive proliferation, angiogenesis, and successful colonization of other tumor cells that have arrived at the site. Indeed, inflammation has been shown to push dormant cells toward re-entry into the cell cycle [18, 80–82], as has age-related senescence in the stromal microenvironment [68].

## Cellular quiescence

Broadly defined, cellular quiescence describes a reversible state of cell cycle arrest. Quiescent cells exit the cell cycle from G1 to enter the reversible G0 phase. In adults, many cells are maintained a quiescent state, including lymphocytes, hepatocytes, dermal fibroblasts, and tissue-resident stem cells in the brain (neural stem cells), skeletal muscle (muscle stem cells or satellite cells), and bone marrow (hematopoietic stem cells) [83]. Careful coordination of quiescence and proliferation in these populations is critical for immunity and for tissue development and repair. T cells, for example, are maintained in a quiescent state until simulation of their T cell receptor by a recognized antigen, upon which they rapidly shift to a proliferative state to fight invading pathogens. Satellite cells are stimulated to exit quiescence upon muscle injury where they proliferate to regenerate damaged tissue. Dysregulation of T cell quiescence (either failure to maintain quiescence or failure to exit quiescence) can impair the immune response. Similarly, dysregulated quiescence in tissue resident stem cells can have damaging effects on tissue homeostasis [83]. Analogous to these tissue resident stem cells, tumors may contain a stem cell-like population that may impact tumor growth, therapeutic resistance, and repopulation [84, 85].

### Cancer stem cells

A subpopulation of stem cell-like quiescent or slow-cycling cells have been identified in multiple cancer types and termed cancer stem cells (CSCs). CSCs from tumors of different tissue origins are phenotypically diverse and are identified by different molecular markers [84], but all display the unifying feature of slow or absent cell division and the presence of tumor-formation capacity [85–87]. Their role(s) in tumor progression are still controversial, but several studies indicate that they are critical drivers of tumor growth [84]. For example, slow-cycling CSCs in melanoma have been demonstrated to be required for tumor maintenance, as when this population is disrupted, serial xenografts fail to proliferate and metastatic outgrowth is hampered [88]. In addition to slow proliferation and tumor formation capacity, CSCs display features of EMT and are therefore of interest in the context of metastatic dormancy and delayed colonization [8–11, 89]. One study demonstrated that ~ 65% of disseminated breast cancer cells in the bone marrow display a CSC phenotype [90]. When compared to the < 10% of these CSCs that are found in the primary tumors, this is strong evidence for a potential role for CSCs in disseminated disease. Regarding delayed relapse, Meng et al. showed that circulating CSCs can be found in the blood of patients who have been disease-free for 20 years, indicating that CSCs may act as a latent reservoir of cells with tumor-forming capacity for late colonization [91].

Another hallmark that is shared by dormant cancer cells and CSCs is the ability to avoid destruction by the immune system [8, 10, 92]. CSCs appear to adopt programs employed by tissue-resident stem cells to avoid immune recognition. One such mechanism is quiescence itself, which promotes the downregulation of antigen presentation, preventing recognition and killing by T cells and natural killer cells [92, 93].

Despite these connections, debate remains over whether CSCs represent a truly dormant population, as populations can be heterogeneous in their proliferative state [10] and have been shown to be proliferative, albeit at slow rates. Therefore, CSCs may more accurately described as ‘slow-cycling’ vs. quiescent [8]. More evidence is needed to determine whether this debate is merely semantic and whether CSCs can play a role in cancer dormancy regardless.

### Quiescence and cancer dormancy

In cancer, “dormancy” and “quiescence” have overlapping, broad definitions — both describe cancer cells that are held in a reversible state of cell cycle arrest — and these terms are therefore commonly used interchangeably. Similar to cancer dormancy, however, the quiescent state is not a monolith. Entry into the quiescent state can be driven by intrinsic cell cycle–related programs or in response to new or newly absent signals from the microenvironment [83]. Induction of G0 arrest is commonly attributed to the actions of cyclin-dependent kinase inhibitors (CDKis), especially p27. Increased p27 activity reduces the ability of CDKs to phosphorylate retinoblastoma protein (Rb), and hypophosphorylated Rb inhibits E2F1 from transcribing target genes necessary for cell cycle progression [83]. Increased p27 expression is commonly used as a marker of quiescence induction, and fluorescent p27 reporters can be used to track live cell entry to and exit from quiescence [94]. Especially *in vivo*, environmental cues are critical in the entry into, maintenance of, and exit from quiescence. Tissue-resident stem cells, for example, reside in specialized niches that contain signals from other cells in the tissue, the extracellular matrix, and soluble factors from local vasculature that drive proliferation or quiescence in a context-dependent manner. A cancer cell also interacts with and responds to its surroundings, which change dramatically upon entrance into the metastatic cascade and eventual deposition in a foreign tissue. Niche factors- both cellular and acellular- in the secondary site may be unfamiliar, and newly present or absent signals from this microenvironment can drive the cell to enter quiescence or promote the maintenance of quiescence if the disseminated cell was previously dormant. Changes to the secondary microenvironment, some driven by the cancer cell itself, may then release the cell into a proliferative state toward eventual colonization and disease relapse [8]. Various recent studies have begun to illustrate just how complex these communication networks are, describing how interactions with immune cells, extracellular matrix components, tissue-specific stromal cells, and environmental factors like oxidative stress can all impact a DTC’s entry into or exit from the dormant state [68, 95–104]. With the increasing understanding that every facet of the cancer cell’s environment impacts its capability to actively divide, it becomes clear that even cells from the same tumor, once disseminated, will have much different fates. The majority will die, but those that survive circulation and extravasation at secondary sites and are destined for a period of dormancy will enter quiescence through different mechanisms depending on the integration of signals from the microenvironment. Even *in vitro*, where quiescence is commonly induced by either contact inhibition, serum deprivation, or loss of adhesion, each of these methods induces G0 arrest through different mechanisms, and the resulting quiescent cells display different gene expression signatures [83, 105–107].

**Fig. 2:**
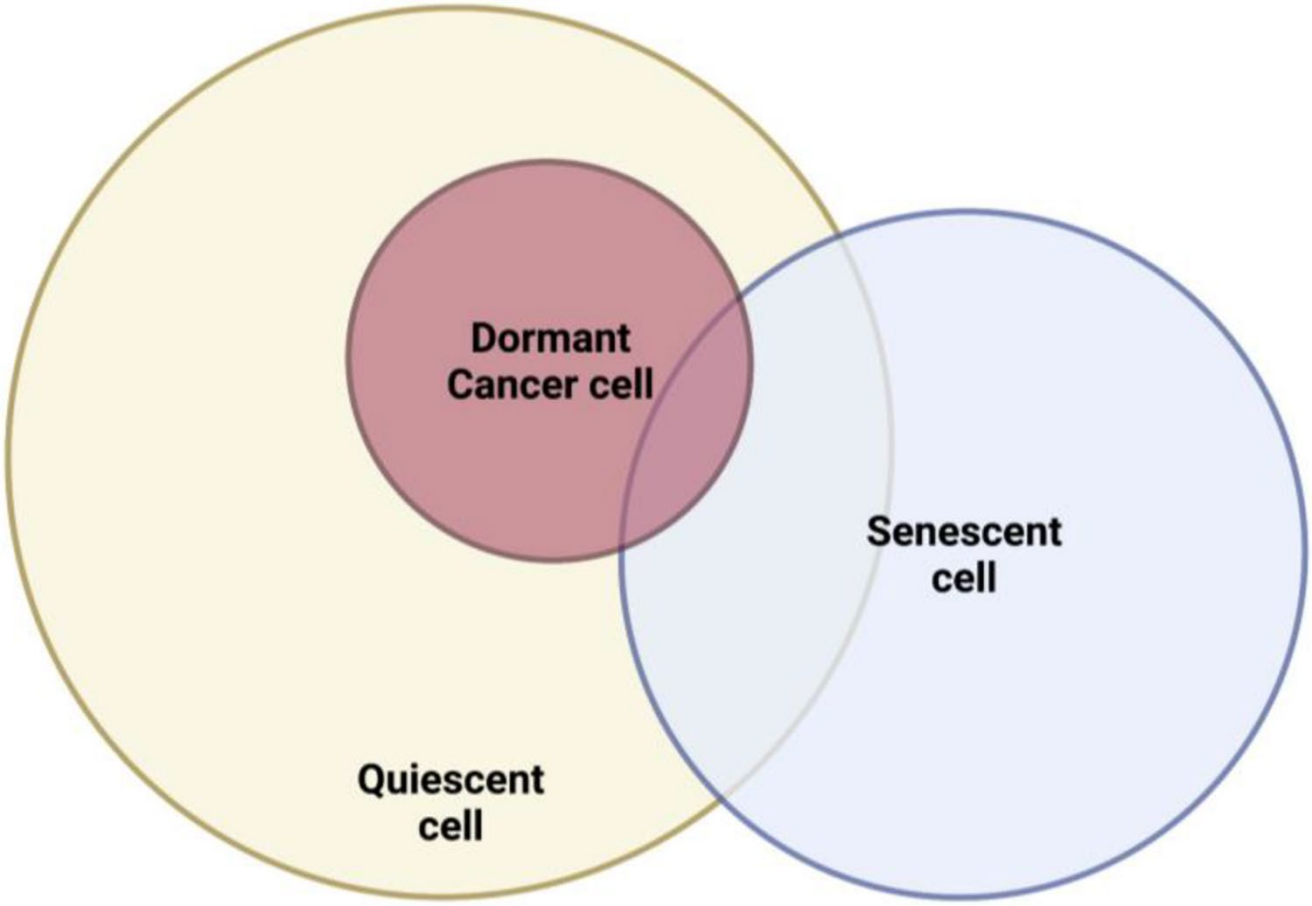
Dormant cancer cells are quiescent but share some characteristics with a senescent cell. Likewise, quiescent and senescent cells share certain characteristics, and these states may exist on a spectrum

In addition to p27, the CDKis p21, p16, and p57 also play a role in quiescence. Interestingly, these are all also implicated in driving senescence. p21, for instance, is a major player in the maintenance of hematopoietic stem cell quiescence and is also considered a canonical marker of senescent cells [35, 81]. Moreover, the transcriptional signatures of quiescent and senescent cells overlap (Table 1) [108]. These common molecular pathways between quiescence and senescence point to the fact that the two states are not wholly unrelated. In fact, cells that have been growth arrested for long periods differ from those arrested for shorter periods in the ‘depth’ of their quiescence. Long-term quiescent cells require more intense growth stimulation and take longer to re-enter the cell cycle [83, 109], and deep quiescence appears to be a transitional state to senescence. The quiescence/deep quiescence/senescence transition is controlled by lysosomal function, which acts as a dimmer switch [110]. These overlaps in phenotype and molecular control indicate that quiescent and senescent states may not be completely binary but lie along a graded spectrum (Fig. 2).

## Conclusions/outlooks

Early dissemination or minimal residual disease remaining after treatment may both lead to reservoirs of dormant cells that fuel eventual metastatic relapse. This period of dormancy offers a unique therapeutic window where intervention could hold these latent cells in their non-proliferative state or eradicate them altogether to halt disease progression before symptoms arise. A better understanding of the biology behind two commonly discussed forms of proliferative arrest—senescence and quiescence—may inform our future thinking about cancer dormancy. Senescence is regularly defined as permanent cell cycle arrest and is therefore often discounted as a player in cancer dormancy. As discussed, some recent evidence suggests that senescence may be reversible in certain contexts, but even if not, it may still play a role in delayed relapse, especially through paracrine signaling from the components of the SASP. Dormant tumor cells are quiescent by definition, but quiescence is not a homogenous cell state. Cells in quiescence come in various forms depending on triggers and microenvironmental cues that can enforce growth arrest or prime cell cycle re-entry. These two forms of cell cycle arrest can present very different phenotypes but are driven by some of the same underlying cell biology and may exist on a quiescence-senescence spectrum as opposed to being binary cell states. Understanding the plasticity and contextual cues that drive quiescence, senescence, and the transitions between them will be key to furthering our understanding of cancer progression.

Targeting cells during the window of cancer dormancy will require advancements in diagnostic tools that improve our ability to identify latent disease before the presence of resistant macroscopic lesions, along with a more thorough understanding of the biology driving the dormant phenotype in these cells. Toward both of these ends, ongoing efforts to define a ‘dormancy signature’ have yielded promising results, though the varying models and contexts in which these studies are carried out may limit their translatability [111–113]. Computational analysis of the combined datasets of these types of studies, such as the one carried out by Uzuner and colleagues [114], may present a way to overcome these limitations and define disease- and context-agnostic drivers of dormancy and recurrence. Further analysis of these datasets may also help to better clarify the relationships between dormancy, quiescence, and senescence. Advances in methods of detecting biomarkers of dormancy and MRD, especially utilizing liquid biopsies for the identification and analysis of circulating tumor cells (CTCs) or circulating tumor DNA (ctDNA) from patients’ blood, have the potential to identify those patients with predisposition toward dormant disease and delayed recurrence based on gene expression signatures which may then help guide subsequent treatment [115–121].

The aforementioned studies largely leverage the rapidly advancing ‘-omics’ technologies, which have been, and will continue to be, critical in providing novel ways to answer questions that were previously unanswerable. The advent of single-cell RNA sequencing (scRNAseq), cytometry by time of flight (CyTOF), matrix-assisted laser desorption/ionization-imaging mass spectrometry (MALDI-IMS), digital special profiling (DSP), and other techniques now make it possible to resolve biological systems spatially (in the case of MALDI-IMS and DSP), and at the single-cell level (with scRNAseq, CyTOF) [122–125]. Coupled to novel molecular technologies such as techniques that enable barcoding and lineage tracing of individual cells, it is also possible to track these biological processes spatiotemporally (reviewed in [126]). As successful metastatic colonization is an inherently rare cellular event, this level of resolution is critical in uncovering transcriptomic signatures of dormancy that may have been hidden in previous bulk RNA sequencing experiments, for example. With lineage tracing, it is possible to then track the fates of each cell expressing a dormancy signature. Tracking differences in these populations during their progression through the metastatic cascade will better elucidate the molecular drivers of both maintenance of the dormant state and the escape from dormancy that drives lethal disease. Elegant studies using these techniques have already dramatically advanced our knowledge of these processes, identifying various cell-intrinsic regulators of dormancy. The processes are wide ranging and include roles for interferon signaling [127]; p38 MAP kinase pathway [128]; an axis including FGF2, the transcription factor ZFP281, and CDH11 [129]; and ECM interactions via COL17A1 [100], to name a few, highlighting just how complex and context-dependent this cell state is. Future work that continues to rely on and advance these techniques will further clarify our understanding of the heterogenous states of tumor dormancy, their relationships to quiescence and senescence, and will be invaluable in detecting dormant cancer cells, uncovering their underlying biology, and defining their role in metastatic relapse.

The end goal of studying the cellular mechanisms of cancer dormancy is to uncover new targets for the prevention of metastatic outgrowth. If this is achieved, then forcing cells to remain dormant or killing them before they outgrow into lethal metastases has the potential to prevent cancer deaths. Despite advancements toward recognizing targetable regulators of cancer dormancy, there are clear challenges in designing human trials that assess the efficacy of treatments targeted to undetectable populations of cancer cells. In addition to the advancement of cancer dormancy biomarkers as discussed, correlating data accumulated from *in vitro* and mouse studies with human data will be critical, and various studies have begun to do just that, opening viable routes toward clinical trials (for example, [130, 131] and well-reviewed in [13, 18]). Beyond these efforts, especially if senescence is reversible in certain contexts, as increasing evidence suggests, targeting senescence to prevent delayed relapse may be more efficient than targeting a quiescent cell, as the senescent phenotype likely offers more specific targets for intervention. Indeed, the development of senolytics (drugs that specifically target senescent cells) is well underway for the treatment of various diseases, including cancer [132]. It has been shown, for example, that clearance of p16-positive senescent cells is possible using genetic perturbation and that it has a positive effect on age-related disorders (though cancer is not assessed) [46]. Since the two states share molecular machinery in many contexts, targeting these overlapping pathways (e.g., intrinsic cell cycle controllers such as CDKis) may have the beneficial effect of eliminating cells in either state. In addition to identifying targetable pathways and developing molecules against them, the development of new animal models that recapitulate disease progression as seen in humans will be critical in advancing our understanding of these processes and how to efficiently target them.

The conflicting fields of senescence and quiescence may not be so conflicting after all, and cells in both states may play a part in cancer dormancy. Quiescence and senescence share some of the same triggers and regulators, so it is likely that cells in both states coexist in the patient and may play complementary roles in leading to delayed metastatic relapse. Utilizing new techniques to resolve these states spatiotemporally and at the single-cell level can detangle complex relationships between quiescent, senescent, and dormant cancer cells and their microenvironments. Understanding the unique programs and their overlaps, along with temporal and microenvironmental contexts, is critical to uncovering targetable pathways and developing treatment paradigms to combat the recurrence of lethal disease.
